# A Data Hiding Technique to Synchronously Embed Physiological Signals in H.264/AVC Encoded Video for Medicine Healthcare

**DOI:** 10.1155/2015/514087

**Published:** 2015-08-25

**Authors:** Raul Peña, Alfonso Ávila, David Muñoz, Juan Lavariega

**Affiliations:** Tecnológico de Monterrey, 64849 Monterrey, NL, Mexico

## Abstract

The recognition of clinical manifestations in both video images and physiological-signal waveforms is an important aid to improve the safety and effectiveness in medical care. Physicians can rely on video-waveform (VW) observations to recognize difficult-to-spot signs and symptoms. The VW observations can also reduce the number of false positive incidents and expand the recognition coverage to abnormal health conditions. The synchronization between the video images and the physiological-signal waveforms is fundamental for the successful recognition of the clinical manifestations. The use of conventional equipment to synchronously acquire and display the video-waveform information involves complex tasks such as the video capture/compression, the acquisition/compression of each physiological signal, and the video-waveform synchronization based on timestamps. This paper introduces a data hiding technique capable of both enabling embedding channels and synchronously hiding samples of physiological signals into encoded video sequences. Our data hiding technique offers large data capacity and simplifies the complexity of the video-waveform acquisition and reproduction. The experimental results revealed successful embedding and full restoration of signal's samples. Our results also demonstrated a small distortion in the video objective quality, a small increment in bit-rate, and embedded cost savings of −2.6196% for high and medium motion video sequences.

## 1. Introduction

Video technology continues to improve the safety and effectiveness in healthcare. Today, physicians and engineers rely on rigorous video-based studies to improve medical practices and procedures. These studies are necessary for the identification of clinical manifestations in patients and for the reduction of errors during medical procedures. Extending the video-based studies to incorporate the analysis of physiological-signal waveforms further enhanced the recognition of clinical manifestations and the reduction of false positive cases. Physicians can rely on simultaneous video-waveform observations to recognize difficult-to-spot signs and symptoms. These observations can also expand the recognition coverage to abnormal health conditions.

The synchronization between the video images and the physiological-signal waveforms is fundamental for enhanced recognition of the clinical manifestations. The identification of signs and symptoms invisible during specific diagnosis is possible with synchronized video-waveform observations. Physicians are able to diagnose seizures in neonates after creating and analyzing a permanent record [1]. The permanent record needed for this diagnosis contains synchronized video and electroencephalographic (EEG) waveforms.

Three commonly used techniques are suitable for video-signal synchronization [2]. The timestamp-based technique is the most common alternative for video-audio synchronization. This technique inserts time codes at each signal stream. These time codes are also useful for future browsing, storage, and reproduction of permanent records [1]. However, the timestamp-based synchronization involves complex tasks such as the capture/compression of the video signal, the acquisition/compression of each physiological signal, the insertion of timestamps into individual streams, and the use of specialized software for stream synchronization [2].

The second technique relies on synchronization marks. The technique sends synchronization marks from a transmission node. The disadvantage of this technique is the need of an additional assistant communication channel to transmit the synchronization mark. The third technique is multiplexing. This technique maintains the correlation among the media streams during the transmission process. However, multiplexing-based synchronization usually results in a loss of agility and integrality.

Data hiding is another alternative for video-waveform synchronization. Data hiding has the goal of embedding information into encoded video sequences with a minimum amount of perceivable degradation [3]. The embedded information can be text, pictures, or physiological-signal samples. Data hiding synchronizes the video and signals after hiding each physiological sample at its corresponding video frame in time.

**Figure 1:**
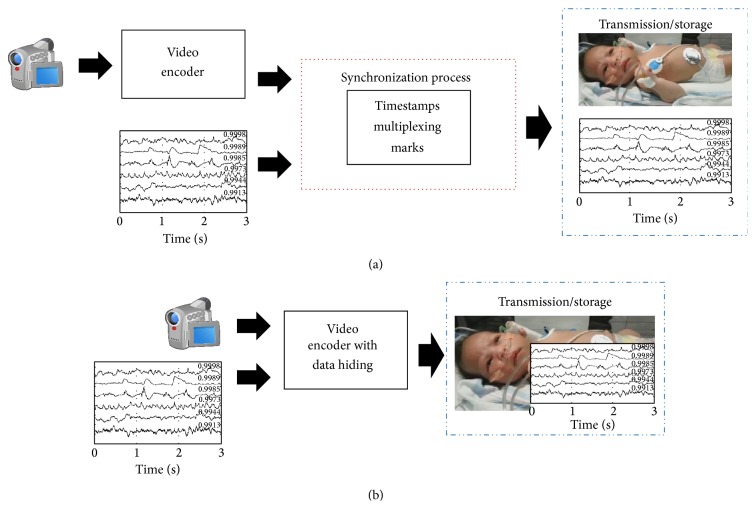
A comparison between (a) conventional synchronization process and (b) our proposed approach.

This paper introduces an improved data hiding technique with a larger data-hiding capacity in the context of medical healthcare. Our data hiding technique synchronously embeds physiological-signal samples into H.264/AVC-encoded video sequences. The implementation of data hiding is simpler than other synchronization techniques. The data-hiding technique requires only one encoder to process video and signal's samples. Data hiding also offers the advantage of a unique communication channel for video-audio transmission and requires no complex tasks related to timestamps, synchronization marks, or multiplexing [4, 5].  Figure 1 illustrates a comparison between the commonly used synchronization processes and our synchronization approach based on data hiding. Other important advantage is that our technique is strongly related to trends in secure handling of medical data during signal transmission. Data hiding makes possible the secure transmission of patient's information over the internet. Important features for secure transmission of personal information are authentication, integrity, and confidentiality [6].

## 2. Background

### 2.1. Data Hiding Techniques

Existing data hiding techniques are able to hide information in the video frames during the video encoding process. In [3], authors proposed a data hiding scheme based on the macroblock's size needed by the H.264/AVC interprediction process. The scheme is able to hide two bits per macroblock and requires the following partitions types: 16 × 16, 16 × 8, 8 × 16, and 8 × 8. The scheme loses no hidden data and may result in bit-rate increments.

In [7], the data hiding scheme relies on constrains associated with the H.264/AVC inter/intraprediction modes. In the interprediction mode, the scheme hides 0 bits at the interprediction mode using the block sizes 16 × 8, 8 × 16, 8 × 4, and 4 × 8. The scheme also hides a 1 bit using the block sizes 16 × 16, 8 × 8, and 4 × 4. In the intraprediction mode, the scheme hides 0 bits using the block sizes 16 × 16 and 4 × 4. Hiding a 1-bit value requires the 8 × 8 block size. The scheme has minimum impact on the video quality and controls the distortion degradation by hiding no data in 4 × 4 blocks.

In [8], the data hiding scheme embeds information during context-based adaptive variable length coding (CAVLC). The scheme hides one bit using the trailing ones parity of the CAVLC code-word. The CAVLC process results in moderate visual degradation and maintains the overall size of the video stream.

In [9], the proposed technique exploited quarter-pixel motion estimation process to hide data. The scheme hides one bit by modulating the best search points of a subblock. The rate-distortion cost is introduced to reduce both the impact on the video quality and the increment in the bit-rate after search point adjustments. The hiding capacity is dependent on the content of the video sequence.

In [10], the authors proposed a data hiding scheme based on an adaptive method. The method hides one bit using the last nonzero coefficient parity after quantization of a 4 × 4 luma block. The scheme relies on an adaptive rather than a fixed point for data embedding. The scheme results in a proportionally direct behavior between the bit-rate and capacity size and between the bit-rate difference and the amount of embedded data.

In [11], the proposed data hiding scheme relies on motion vectors and mode selection. The scheme only hides one bit per frame. This scheme embeds data using the macroblock search regions with a left area restriction for a 1 bit and a right area restriction for 0 bits.

In [4, 5], the authors demonstrated that existing data hiding schemes in [8, 10] successfully embedded audio into encoded video sequences with minor impact on video image quality and bit-rate.

The desirable features of a data hiding technique suitable for embedding physiological signals into encoded video sequences are a large data hiding capacity, a low impact in video quality, and a minor effect in bit-rate of the video.  Table 1 compares the data hiding schemes previously reviewed considering three metrics: hiding data capacity, the maximum PSNR_*Y*_ (objective video quality), and bit-rate distortion. These schemes offer relatively low values for the three metrics. Therefore, the main limitation resides in the data hiding capacity. In [9], the quarter-pixel motion estimation scheme offers the highest data hiding capacity using an 8 × 8 partition. This scheme also offers very low PSNR_*Y*_ distortion and less than 1.32% of bit-rate distortion. Our proposed technique extends the quarter-pixel motion estimation scheme to satisfy the data hiding capacity needs and to ensure the low PSNR_*Y*_ and bit-rate distortions.

### 2.2. Motion Estimation in H.264/AVC

**Figure 2:**
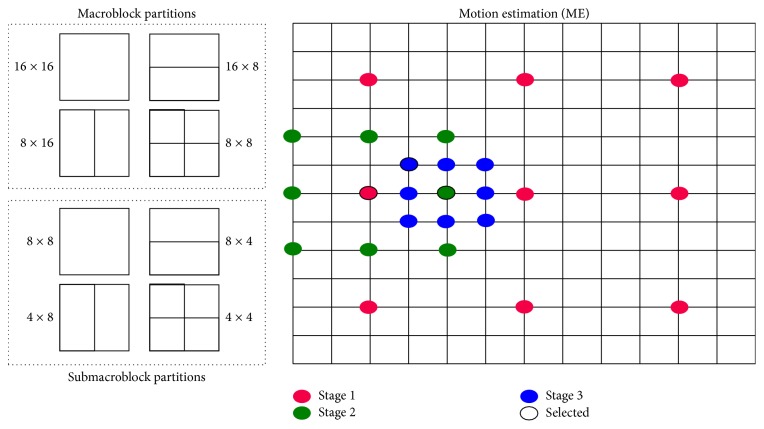
Macroblock partitions and search points in motion estimation.

Motion estimation (ME) is an important element in the H.264/AVC interprediction process. For a given frame, the ME goal is to find the best predictions for both levels: macroblock (MB) selection and motion vector (MV) estimation. A MB is an array of 16 × 16 pixels. The MB selection process assumes the partitioning illustrated in Figure 2. Each partition contains a MV value. Equation (1) shows how to select the best block partition by calculating the Lagrangian rate distortion (*J*_mode_) optimization. In this equation, *λ*_mode_ is the Lagrangian multiplier, SSD is the sum of the squared difference between the original and the reconstructed block, and *R* is the number of bits of MB parameters such as quantization parameter, header, motion vectors, and residue coefficients:

(1) $$\begin{array}{c} J_{\text{mode}}=\text{SSD}+\left(\lambda_{\text{mode}}\right)\left(R\right). \end{array}$$

The motion estimation process computes motion vectors for each macroblock partition found in each video frame. At a given frame, the ME process searches for the new MB position of each MB located in the reference frame. The ME process calculates motion vectors based on these new MB positions and encodes these vectors in the encoded frames. Figure 2 illustrates the three ME stages to compute a MV. The first ME stage identifies the best MB position at the integer-pixel mesh. The second ME stage identifies the best MB position at the half-pixel mesh based on the best integer-pixel position. The third ME stage identifies the best MB position at the quarter-pixel mesh based on the best half-pixel position. The selected position becomes the final MV value.

### 2.3. Application Examples of Video-Based Medical Care

This section presents additional examples of medical applications related to the simultaneous observation of video and physiological-signal waveforms.  Table 2 shows the names of the applications and the specific physiological signals needed for simultaneous correlation with the video. In [1], physicians take advantage of synchronized EEG recordings with video to correlate clinical manifestations such as lip smacking, fixing of eyeballs, and cyclic leg movements.

In [12], physicians take advantage of synchronized digital video recordings to identify nocturnal breathing anomalies usually undetected by standard polysomnography. Successful identification of these anomalies requires the correlation among EEG recordings, oxygen saturation (SpO_2_), endtidal CO_2_ level, in-video leg movement, and in-video rapid eye movement (REM).

In [13], the purpose is to assess the validity of a new physical-activity monitor in the context of congestive heart failure. This monitor utilizes body-fixed accelerometers to distinguish among activities such as body postures, sitting, standing, normal walking, stairs walking, cycling, and wheelchair driving. These in-video activities are correlated with the accelerometer waveforms to assess the correct operation of the activity monitor.

In [14], clinical investigators perform rigorous studies to enhance patient safety in operating rooms. The investigators first elaborate a permanent feedback record containing the in-video health delivery process, vital signs, and other signals. Then, the investigators reproduce this permanent record to observe and to assess the health delivery process. The synchronization of the video and the physiological-signal waveforms is fundamental for the identification of factors resulting in adverse events.

## 3. The Proposed Data Hiding Technique

**Figure 3:**
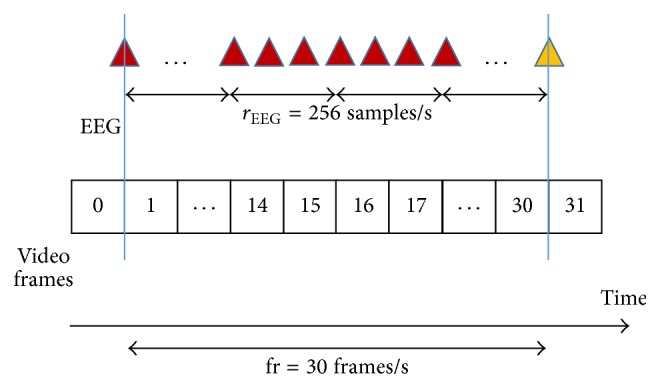
Synchronization process.

Our proposed technique hides streams of data samples into encoded video sequences. The implementation of our technique is a set of software routines added to the original H.264/AVC codec.  Figure 3 illustrates the synchronization between the video and the physiological-signal waveforms of EEG samples. Our technique synchronizes video and EEG signals by hiding samples of these signals at their corresponding frame in time.

### 3.1. Encoding Process in Our Data Hiding Technique

Our technique modifies the H.264/AVC interprediction process to hide the samples of the physiological signals. Our technique relies upon the quarter-pixel motion estimation process, part of the H.264/AVC encoder, to hide these samples into encoded video frames. Our technique divides the search points into four groups to improve the data hiding capacity of the original technique [9]. Equation (2) presents the proposed partitioning of search points and the binary assignment for each partition. The expression in (3) indicates how to select the search point in each group with the minimum distortion cost. In (3), *Ji* is the Lagrangian rate distortion parameter:

(2) $$\begin{array}{c} \text{searchpos}\in \left\{\begin{array}{cc} G1=\left\{5,6\right\} & \text{if}\;\;“00\text{”}\\ G2=\left\{1,2\right\} & \text{if}\;\;“01\text{”}\\ G3=\left\{3,4,7,8\right\} & \text{if}\;\;“10\text{”}\\ G4=\left\{0\right\} & \text{if}\;\;“11\text{”}, \end{array}\right. \end{array}$$

(3) $$\begin{array}{c} \text{searchpos}=\left\{i\;|\;\min\left(Ji\right),\;i\in \left(G1,G2,G3,G4\right)\right\}. \end{array}$$

**Figure 4:**
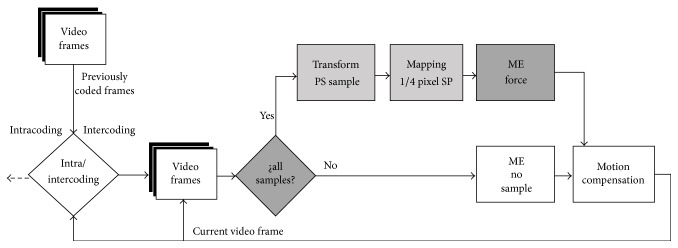
The block diagram of our proposed data hiding technique based on H.264/AVC encoder.

Our proposed technique, illustrated in Figure 4, repeats the gray blocks until no samples are available. The blocks in gray are the additional routines needed to implement our proposed data hiding algorithm. The alternate path executes the original H.264/AVC motion estimation process.  Algorithm 1 presents details of our data hiding algorithm for encoding. Our technique hides the signal samples in the motion vectors of each block partition located at a frame. Each motion vectors hides two bits of a signal sample. Our technique hides no samples in the block types I4 MB and I16 MB due to their association with intraframe prediction. Our technique is also unable to hide samples into PSKIP blocks due to the lack of motion vectors.

Our technique also incorporates an approach to overcome the data-hiding capacity limitation found in low-motion video sequences. PSKIP blocks are the most common block partition found in encoded low-motion video sequences. A large number of PSKIP blocks limit the data hiding capacity of our technique due to few number of motion vectors found in the low-motion video sequence. Therefore, our proposed technique forces the H.264/AVC encoder to replace the PSKIP block by a P16 × 16 block partition. This PSKIP replacement adds a motion vector to the data hiding capacity of our technique. The PSKIP replacement also contributes to maintaining the synchronization between the encoded video and the physiological signals. However, this replacement may also result in an increment in the bit-rate of the sequence.  Algorithm 2 presents details of the algorithm for low-motion sequences.

### 3.2. Decoding Process of Our Data Hiding Technique

**Figure 5:**
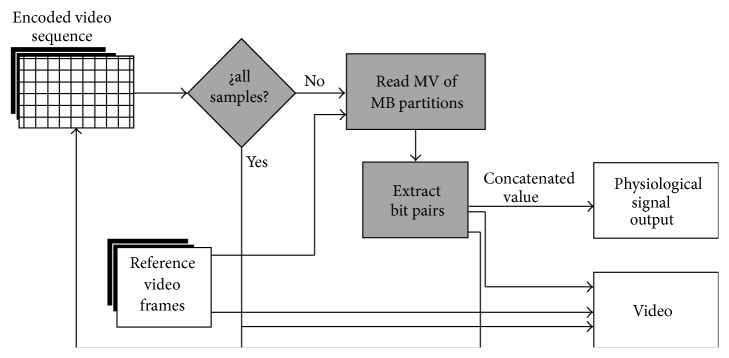
Sample extraction during the decoding process.

Our data hiding technique extracts the samples of the EEG signal; at the same time, conventional video H.264/AVC decoding process takes place. This is an important feature at the time to do synchronized playback of both video and physiological-signal waveforms. The sample extraction algorithm in our technique catches and gathers sample data. This extraction algorithm, illustrated in Figure 5, is repeated as many times as needed to extract all the samples embedded in the video sequence. To do this, our data hiding technique interacts with the H.264/AVC decoding process.

The routine reads the motion vector (MV) of every macroblock partition and inputs the MV_*x*_ and MV_*y*_ components into (4) to identify a binary combination. The |MV | %2 terms output the quarter-pixel search point values. The routine obtains the bit-pair samples after identifying the *G*1 to *G*4 values mapped into (2):

(4) $$\begin{array}{c} \text{type}\in \left\{\begin{array}{cc} G1 & \text{if}\;\;\left(\left|{\text{MV}}_{x}\right|\%2=1\right),\;\;\left(\left|{\text{MV}}_{y}\right|\%2=0\right)\\ G2 & \text{if}\;\;\left(\left|{\text{MV}}_{x}\right|\%2=0\right),\;\;\left(\left|{\text{MV}}_{y}\right|\%2=1\right)\\ G3 & \text{if}\;\;\left(\left|{\text{MV}}_{x}\right|\%2=1\right),\;\;\left(\left|{\text{MV}}_{y}\right|\%2=1\right)\\ G4 & \text{if}\;\;\left(\left|{\text{MV}}_{x}\right|\%2=0\right),\;\;\left(\left|{\text{MV}}_{y}\right|\%2=0\right). \end{array}\right. \end{array}$$

Algorithm 3 presents details of our proposed decoding algorithm. This decoding process extracts the physiological samples from the encoded video sequences.

## 4. Results

**Figure 6:**
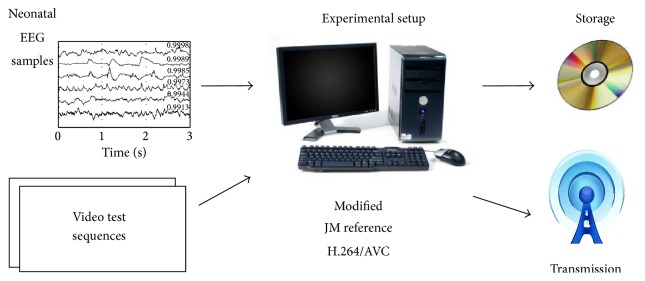
Experimental setup for our synchronization data hiding scheme.

The experimental setup, illustrated in Figure 6, included a PC, an EEG database, and a set of seven video test sequences. The EEG samples were extracted from the CHB-MIT Scalp EEG Database. This database is located at the PhysioBank digital recordings (http://www.physionet.org). The experimental setup included 6 signal electrodes at 256 samples per second and 12-bit sample resolution [1]. The EEG samples generated in one second were embedded into the first 30 frames of each test video sequence to establish synchronization.

The implementation of encoding and decoding processes needed the modification of the JM reference software version 16.1.  Table 3 shows the JM configuration. A program was developed to provide and convert samples from the database to the encoder.

The video test sequences had a CIF (352 × 288) resolution and a 4:2:0 YUV format. The name of the test sequences are akiyo, bridge-far, carphone, football, foreman, mobile, and neonatal. Neonatal is not considered a standard video test sequence. Neonatal was introduced to match the context of the application example related to EEG seizures on neonates. The selected coding structure of the bit stream is “IPPP…” to have an intraframe encoded in the first frame and interframes encoded in the remaining frames.

Metrics to evaluate the effectiveness of our proposed technique are video objective quality, bit-rate difference, embedding cost, and perceptual quality of the image. The peak signal-to-noise ratio (PSNR), illustrated in (5), is an objective quality metric to report video image degradation. *M* and *N* are the height and the width of the video frame, respectively. *Iij* and *Iij*′ represent the original pixel and the processed pixel, respectively [5]. The PSNR_*Y*__diff_ metric, illustrated in (6), indicates how the luma (*Y*) samples impact the video quality after embedding the physiological samples. PSNR_*Y*_′ represents the impact of luma samples generated by embedding the samples, and PSNR_*Y*_ represents the impact of luma samples generated with the original H.264/AVC encoder:

(5) $$\begin{array}{c} \text{PSNR}=10\log\frac{\max\left(Iij^{2}\right)}{\left(1/M\right)\sum_{i}^{M}\sum_{j}^{N}{\left[Iij-Iij^{\prime }\right]}^{2}}, \end{array}$$

(6) $$\begin{array}{c} {{\text{PSNR}}_{Y}}_{\text{diff}}={\text{PSNR}}_{Y}^{\prime }-{\text{PSNR}}_{Y}. \end{array}$$

Equation (7) shows how to calculate the change of bit-rate (BRI). *R* is the original bit-rate and *R*′ is the embedding samples bit-rate. Equation (8) shows how to estimate the embedded cost Oe, Ov is the data volume generated by the original video coder, DHe is the data volume generated by embedding the samples, and EEGe is the embedded data volume [7]. The EEGe term refers to the data coming from the EEG signals. The perceptual quality provides an estimation of subjective quality of the image obtained by visual inspection:

(7) $$\begin{array}{c} \text{BRI}=\frac{R^{\prime }-R}{R}\times 100\%, \end{array}$$

(8) $$\begin{array}{c} \text{Oe}=\frac{\text{DHe}-\text{Ov}-\text{EEGe}}{\text{Ov}+\text{EEGe}}\times 100\%. \end{array}$$

**Figure 7:**
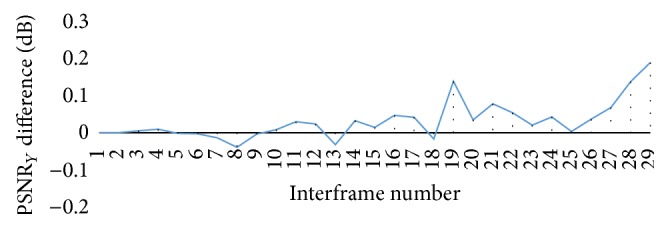
PSNR luma difference values for neonatal video sequence.

Figure 7 compares the PSNR_*Y*_ difference between the original and the embedding data encoding processes. The graph shows a very small difference between PSNR_*Y*_ values for the first 30 frames of the neonatal video sequence. The largest increment in quality was 0.187 dB. This increment appeared in the 29th frame. The largest decrement in quality was −0.039 dB. This decrement appeared in the 8th frame.

**Figure 8:**
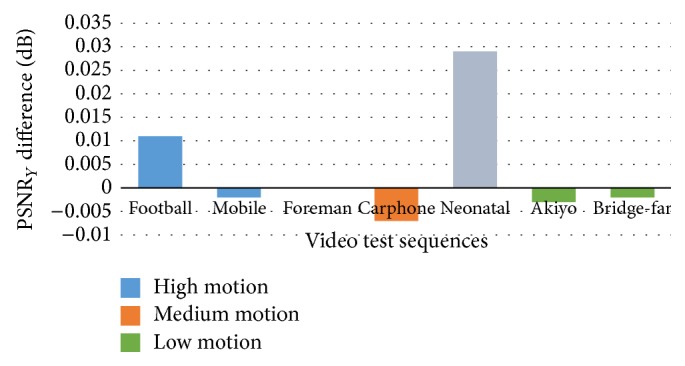
PSNR luma difference for seven video test sequences.

Figure 8 demonstrates that the embedding process has minor effect on the video objective quality of seven test sequences. The *y*-axis represents the PSNR luma difference values expressed in decibels. The positive values above average, 0.011 dB and 0.029 dB, represent a very small improvement in video quality. The negative values, from −0.002 dB to −0.007, represent a small degradation in video quality. These degradations and improvements are due to the lack of bit-rate constraint.

**Figure 9:**
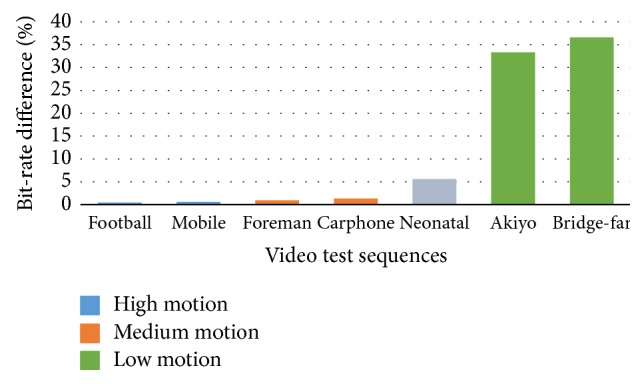
Change of bit-rate for seven video test sequences.

Embedding the EEG data into the video sequences produced increments of bit-rate for the seven video test sequences as illustrated in Figure 9. Our experimental setup included high motion, medium motion, and low motion video test sequences. The amount motion of the neonatal video sequence was considered between medium and low. The graph presents the neonatal sequence with gray color to indicate that it is not a standard video test sequence. For the high and medium motion sequences, there are small bit-rate increments. The bit-rate increments ranged from 0.45% to 1.34%. For the neonatal and low motion sequences, the PSKIP block replacement occurred and resulted in bigger bit-rate increments. The bit-rate increments ranged from 5.6% to 36.58%. However, the changes in the bit-rate had no effect on the video-waveform synchronization.

Table 4 shows the results for the seven test sequences in terms of embedded capacity, modified macroblocks, modified motion vectors, PSNR_*Y*_ difference, bit-rate, and the embedded cost. For the high, medium motion and neonatal sequences, the embedded cost ranged from −0.1273% to −2.6196% representing savings in data volume. For the low motion sequences, the embedded cost had an increment in 15.8726% and 19.3017%. These results indicate that our data hiding technique offers both an adequate efficiency for video-signal transmission and savings in the storage of high, medium, and neonatal sequences.

**Figure 10:**
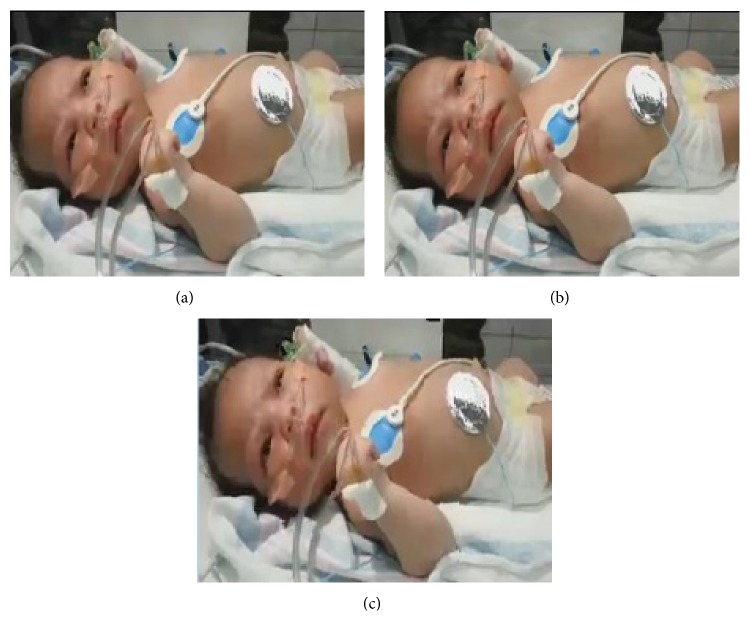
10th frame of neonatal video sequence. (a) Original image, (b) image using video original coding, and (c) image with EEG data.

The inspection of subject quality indicated minimum visual artifacts or distortion between original and data-hiding images.  Figure 10 illustrates the perceptual quality of the video frames for the 10th frame of the neonatal sequence. Thus, data hiding generates no significant difference in quality from a human eye perception.

Our technique will offer an adequate performance in the context of the application examples presented in Section 2.3. The video sequences of these applications exhibit sufficient amount of motion. In the neonatal-seizures application, a sufficient amount of motion is needed to identify clinical manifestations like epileptic attacks. In the application about breathing disorders, respiratory and abnormal movements are needed for accurate diagnosis. In the application about congestive heart failures, the motion is associated with the physical activities of the patient. Finally, the amount of motion is associated with the medical staff activity rather than the patient for the application example related to the improvement of medical practices in operating rooms.

## 5. Conclusions

The proposed data hiding technique was demonstrated to be suitable for the medicine healthcare context. Our technique successfully embedded samples of six EEG signals into encoded video sequences with high, medium, and low motion. Our technique also extracted the hidden samples from the encoded video sequences without loss of information. The implementation of our technique required simpler tasks compared to other existing synchronization techniques: (1) less number of encoders and decoders, (2) no timestamps needed, (3) no software needed for synchronization of video and signal streams, and (4) higher data capacity compared to other data hiding techniques, especially for high motion sequences.

The experimental results demonstrated minimum degradation in video quality and data savings in terms of storage/transmission. The experimental results for high and medium motion video test sequences ranged from −0.007 dB to 0.011 dB in PSNR luma difference, from 0.4459% to 1.3446% in the bit-rate difference, and from −2.6196% to −0.1273% in embedded cost. The changes in PSNR_*Y*_ difference, and bit-rate resulted in both no impacts in video-waveform synchronization and minimum distortions in video quality. For storage and transmission purposes, the embedded cost for high and medium motion video sequences represent savings. For low motion video sequences the experimental results ranged from –0.003 dB to −0.002 dB in PSNR_*Y*_ difference, from 33.3143% to 36.575% in the bit-rate difference, and from 15.8725% to 19.3017% in embedded cost. The changes in bit-rate were higher compared to the high and medium video sequences.

## Figures and Tables

**Algorithm 1:**
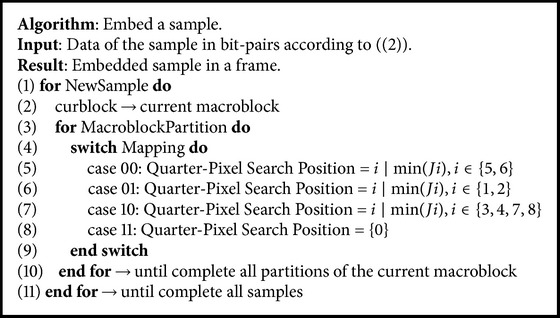
Algorithm of our data hiding encoding technique.

**Algorithm 2:**
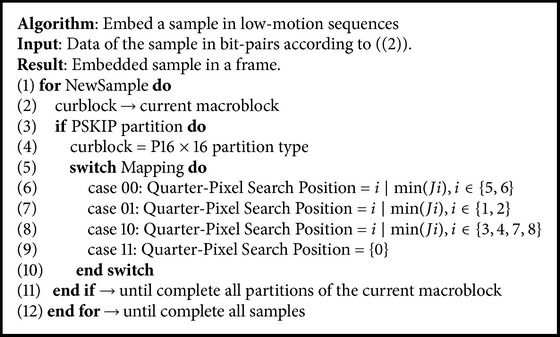
Algorithm of our data hiding encoding technique for low motion sequences.

**Algorithm 3:**
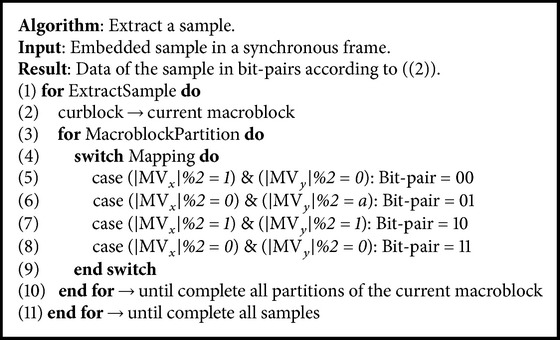
Algorithm of our data hiding decoding technique.

**Table 1:** Qualitative metrics and statistics of the data hiding (DH) schemes reviewed.

Reference	DH schemes	DH capacity	Max. PSNR_*Y*_ distortion	Max. bit-rate distortion
[3]	Forcing block-type partitions	2 bits per MB (interframes)	−1.4 dB (40 kbps)	7000 bps
[7]	Grouping block-type partitions	1 bit per MB (inter/intraframes)	−0.09 dB	4.65%
[8]	Parity of trailing ones CAVLC	1 bit per MB (intraframes)	−2.57 dB	0.039%
[9]	Quarter-pixel search positions	1–4 bits per MB (interframes)	−0.03 db	1.31%
[10]	Last nonzero coefficient parity	1 bit per MB (interframes)	Approx. −0.01 dB	1200 bps
[11]	Motion vectors and mode sel.	1 bit per frame (interframes)	0.03 dB	0.98%

**Table 2:** Examples of medical applications.

Reference	Medical application	Phys. signals
[1]	Silent neonatal seizures	EEG
[12]	Sleep-related breathing disorders	EEG, ECG, SpO_2_, and CO_2_
[13]	Physical activity and congestive heart failure	Accelerometers
[14]	Patient safety in anesthesia operating rooms	Vital signs

**Table 3:** Configuration parameters for JM reference software.

Parameter	Resolution
Profile	Baseline
Frames	30
Motion estimation algorithm	Full search
RD optimization and rate control	Disabled
8 × 8 subblocks	Disabled
Number of reference frames	1
Quantization parameter (QP)	28

**Table 4:** Experimental results for seven video test sequences.

Video sequences	Embedded capacity (bits)	Modified MBs	Modified QP-MV	PSNR_*Y*diff_ (dB)	BRI (%)	Oe (%)
Football	45598	0	6957	0.011	0.4459	−0.3787
Mobile	43598	0	7017	−0.002	0.5837	−0.1273
Foreman	28550	0	6702	0	0.9341	−2.6196
Carphone	23454	0	6699	−0.007	1.3446	−2.2459
Neonatal	16544	944	6364	0.029	5.604	−1.1826
Akiyo	5718	6357	5145	−0.003	33.3143	15.8725
Bridge-far	3650	7391	4662	−0.002	36.575	19.3017
